# Hypofractionated stereotactic body radiation therapy as monotherapy for intermediate-risk prostate cancer

**DOI:** 10.1186/1748-717X-8-30

**Published:** 2013-01-31

**Authors:** Andrew W Ju, Hongkun Wang, Eric K Oermann, Benjamin A Sherer, Sunghae Uhm, Viola J Chen, Arjun V Pendharkar, Heather N Hanscom, Joy S Kim, Siyuan Lei, Simeng Suy, John H Lynch, Anatoly Dritschilo, Sean P Collins

**Affiliations:** 1 Department of Radiation Medicine, LL Bles Building, 3800 Reservoir Rd NW, Washington, DC 20007, USA; 2 Department of Biostatistics, Bioinformatics, and Biomathematics, Georgetown University, Washington, DC, USA; 3 Department of Urology, Georgetown University Hospital, Washington, DC, USA

**Keywords:** Stereotactic body radiotherapy, Prostate cancer, SBRT, CyberKnife, Intermediate-risk, Monotherapy, Hypofractionation

## Abstract

**Background:**

Hypofractionated stereotactic body radiation therapy (SBRT) has been advanced as monotherapy for low-risk prostate cancer. We examined the dose distributions and early clinical outcomes using this modality for the treatment of intermediate-risk prostate cancer.

**Methods:**

Forty-one sequential hormone-naïve intermediate-risk prostate cancer patients received 35–36.25 Gy of CyberKnife-delivered SBRT in 5 fractions. Radiation dose distributions were analyzed for coverage of potential microscopic ECE by measuring the distance from the prostatic capsule to the 33 Gy isodose line. PSA levels, toxicities, and quality of life (QOL) measures were assessed at baseline and follow-up.

**Results:**

All patients completed treatment with a mean coverage by the 33 Gy isodose line extending >5 mm beyond the prostatic capsule in all directions except posteriorly. Clinical responses were documented by a mean PSA decrease from 7.67 ng/mL pretreatment to 0.64 ng/mL at the median follow-up of 21 months. Forty patients remain free from biochemical progression. No Grade 3 or 4 toxicities were observed. Mean EPIC urinary irritation/obstruction and bowel QOL scores exhibited a transient decline post-treatment with a subsequent return to baseline. No significant change in sexual QOL was observed.

**Conclusions:**

In this intermediate-risk patient population, an adequate radiation dose was delivered to areas of expected microscopic ECE in the majority of patients. Although prospective studies are needed to confirm long-term tumor control and toxicity, the short-term PSA response, biochemical relapse-free survival rate, and QOL in this interim analysis are comparable to results reported for prostate brachytherapy or external beam radiotherapy.

**Trial registration:**

The Georgetown Institutional Review Board has approved this retrospective study (IRB 2009–510).

## Background

The treatment of prostate cancer has evolved to include optimization of radiation dose distributions and radiobiological effectiveness. Clinical evidence suggests that the α/β ratio of prostate cancer is perhaps as low as 1.5-2 Gy [1]. Given an α/β ratio for prostate cancer that is less than the generally accepted value of 3 Gy for late rectal complications, the linear-quadratic model predicts a greater therapeutic gain for hypofractionated courses of radiotherapy over conventionally fractionated treatment regimens. Early experience with investigations of limited hypofractionation (fraction sizes from 2.5 to 3.5 Gy) has revealed that such regimens are effective without undue toxicity [2]. One Phase III trial has shown comparable toxicities and improved freedom from biochemical failure with a hypofractionated treatment regimen of 62 Gy in 20 fractions compared to a conventionally fractionated regimen of 80 Gy in 40 fractions [3]. The linear-quadratic model predicts that even larger fraction sizes may provide additional improvements in control.

Large radiation fractions have been successfully used as a boost to escalate the dose to the prostate and seminal vesicles (SV). The hypofractionated boost is used to supplement a course of conventionally fractionated external-beam radiotherapy (EBRT) designed to treat a larger volume encompassing the microscopic disease adjacent to the prostate and seminal vesicles. Studies investigating a high dose-rate (HDR) brachytherapy boost delivering 4–11.5 Gy/fraction over 2 to 4 sessions combined with EBRT report five-year biochemical control rates in excess of 85% for intermediate-risk prostate cancer patients with acceptable toxicities [4]. The tight margins and steep dose gradients delivered with HDR brachytherapy can be approximated with SBRT using the CyberKnife (CK) system, [5,6] which uses real-time image guidance to account for intrafraction prostatic motion [7]. EBRT with an SBRT boost has been explored in intermediate-risk prostate cancer with favorable early results [8,9].

Delivering an entire course of radiotherapy for prostate cancer using large fractions rather than as a boost to a course of conventionally fractionated EBRT could be radiobiologically advantageous. The safety, efficacy, and convenience of such monotherapy with hypofractionated courses of treatment at 6–9.5 Gy per fraction has been demonstrated for localized prostate cancer using HDR brachytherapy [10-15]. Initial published outcomes for hypofractionated SBRT using the CK system as monotherapy for patients with clinically localized prostate cancer show PSA responses and toxicity profiles that are historically comparable to conventional external beam radiotherapy or HDR brachytherapy [16-24]. However, with the exception of the recent paper by Lee *et* al., the majority of the patients in these studies have low-risk disease, and there is a concern that the tight margins required to limit the normal tissue doses to the rectum and bladder in hypofractionated radiotherapy may not be adequate to treat the microscopic disease from extracapsular extension (ECE) and SV involvement that is present in 35–50% of intermediate-risk patients [25]. Here, we provide dosimetric data to support the adequacy of such treatment and report the early outcomes for intermediate-risk prostate cancer patients treated with hypofractionated SBRT monotherapy using the CK system.

## Methods

This retrospective review of prospectively collected data from 41 consecutively treated patients receiving hypofractionated stereotactic body radiotherapy at Georgetown University Hospital as monotherapy for histologically-confirmed intermediate-risk prostate cancer. Intermediate risk was defined using the National Comprehensive Cancer Network (NCCN) criteria of patients with at least one of the following risk factors: Stage T2b - T2c disease, a Gleason score of 7, or a PSA of 10 – 20 ng/mL [26]. Clinical stage was assigned according to the 6th edition of the American Joint Committee on Cancer definitions. Exclusion criteria included less than one year of clinical follow-up, clinical involvement of lymph nodes or distant metastases on pre-treatment imaging, prior prostate cancer-directed therapy, or prior pelvic irradiation. Patients who received androgen deprivation therapy (ADT) were excluded. Institutional review board approval was obtained for this analysis.

### Treatment planning and delivery

The fiducial placement and CT/MRI simulation procedures have been previously described in Lei *et al*. [27] The gross tumor volume (GTV) was defined as the prostatic capsule and the proximal seminal vesicles up to the point that the left and right seminal vesicles separated. To create the clinical target volume (CTV), this volume was further expanded 3 mm posteriorly and 5 mm in all other directions to cover areas of potential ECE. The planning target volume (PTV) was equal to the CTV. No attempt was made to spare the neurovascular bundles in treatment planning. Treatment planning was performed using Multiplan (Accuray Inc., Sunnyvale, CA). The prescription dose was either 35 or 36.25 Gy to the PTV in 5 fractions over 1–2 weeks. Patients with an elevated pre-treatment AUA scores, large prostates, or medical co-morbidities were prescribed the lower dose [20]. The target doses and dose constraints have been described in Oermann *et al*. [28]

**Figure 1:**
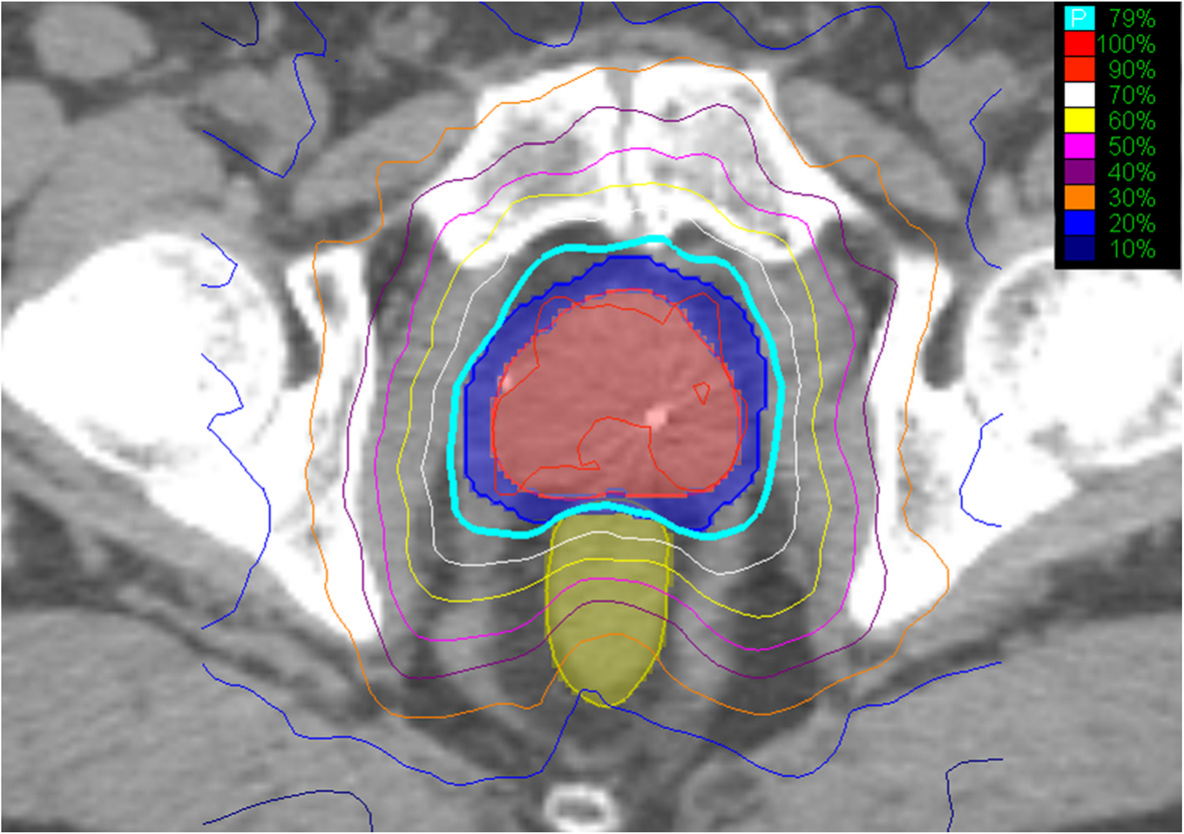
**Example treatment plan showing an axial view.** The volumes represent the GTV (red), PTV (blue) and rectum (light green). The prescription isodose line (79%) is denoted by the thick light blue line.

During treatment, paired orthogonal x-rays were taken every 3–6 beams to adjust for the translational and rotational intrafraction movement of the prostate, with the goal of keeping the translational corrections between images to less than 2 mm, and the rotational corrections to less than 5 degrees. This corresponds to approximately every 30–60 seconds at the start of treatment, and the time between images is varied during the course of treatment depending on amount of prostate motion [7,27]. All plans were reviewed and approved by a single experienced physician in our institution (S.C.). Figure 1 shows an axial view of a typical treatment plan. Quality assurance for the SBRT plans was assured by a weekly departmental review of cases.

### Pretreatment assessment and follow-up

A clinical examination including a digital rectal exam (DRE), a PSA level, and a quality of life (QOL) questionnaire were performed prior to the initiation of stereotactic body radiotherapy, at 1 and 3 months post-treatment, and every 3 months thereafter. The QOL questionnaires evaluated urinary, bowel, and erectile functions using the International Prostate Symptom Score (IPSS), [29] the Expanded Prostate Cancer Index Composite (EPIC) short form, [30] and the 12-item Medical Outcomes Study Short Form (SF-12) version 2 questionnaires [31]. Acute and late toxicities were scored using the Common Terminology Criteria for Adverse Events (CTCAE) Version 4.0. Acute toxicity is defined as occurring within 6 months of completing treatment, and late toxicity as those events occurring later than 6 months. Biochemical failure was defined as a PSA rise of ≥ 2 ng/mL above the nadir [32].

### Dosimetric assessment

The coverage of potential extracapsular extension of disease with a dose sufficient to control gross and microscopic disease was the focus of evaluation. The distance from the GTV to the 33 Gy isodose line of the treatment plans was measured in 45 degree increments on 3 axial planes: the plane 1 cm caudal from the base of the prostate, the plane 0.5 cm cranial from the apex, and the plane in the mid-prostate halfway between the 2 other planes. This dosimetric analysis has been previously described in brachytherapy implants [33,34]. The 33 Gy isodose line was chosen as the dose that would treat potential ECE. Conservatively assuming an α/β ratio of 3 Gy, the linear-quadratic equation would predict that this corresponds to a dose of 66 Gy delivered in 1.8 Gy daily fractions, which is the dose considered sufficient for treating microscopic residual disease in the prostatic fossa after prostatectomy [35]. In our SBRT study 33 Gy is greater than 90% of the prescription dose.

### Statistical analysis

Descriptive statistics were used to summarize the patients’ baseline clinical characteristics, the dosimetric characteristics, as well as the rates of observed toxicities. QOL data from time points in which more than 80% of patients completed the questionnaires were included in the analysis. An QOL change of one-half standard deviation (SD) from the baseline QOL score, defined as the minimal important difference (MID), was used to denote a clinically significant change in the QOL score [36]. The two-sided paired Wilcoxon rank-sum test was used to calculate the significance of differences in the mean scores on follow-up as compared to the baseline values. Parameters were identified as significant if the two-tailed p-value was less than 0*.*05. MedCalc® version 11.6.1.0 was used for the statistical analyses.

## Results

### Patients

Forty-one men were treated between October 1, 2008 and October 30, 2009 met the inclusion criteria. Their baseline characteristics are summarized in Table 1. The median clinical follow-up is 21 months (range 13 – 27.5 months). Eight patients received a total dose of 35 Gy, and 33 patients received 36.25 Gy. The median prescription isodose line was 77% (range 75%–80%), which covered a median 95.1% of the PTV (range 94.2%–96.4%). The dosimetric constraints to normal tissues were met in the majority of patients.

**Table 1:** Patient characteristics

**Number of patients**	**n = 41**	
**Age**	***Median***	***Range***
	69	60–92
**Race**	***n***	***%***
Caucasian	26	63%
African-American	15	37%
**Prostate volume (cc)**	***Median***	***Range***
	37	20–63
**PSA (ng/mL)**	***Median***	***Range***
	6.9	3.5–18.3
**T Stage**	***n***	***%***
1b	1	2%
1c	28	68%
2a	6	15%
2b	4	10%
2c	2	5%
**Gleason score**	***n***	***%***
3+3	9	22%
3+4	23	56%
4+3	9	22%
Number of risk factors*	***n***	***%***
1	34	83%
2	7	17%
3	0	0%

* Factors include PSA 10–20 ng/mL, Gleason 7, or T2b-2c.

### Dosimetric analysis

**Figure 2:**
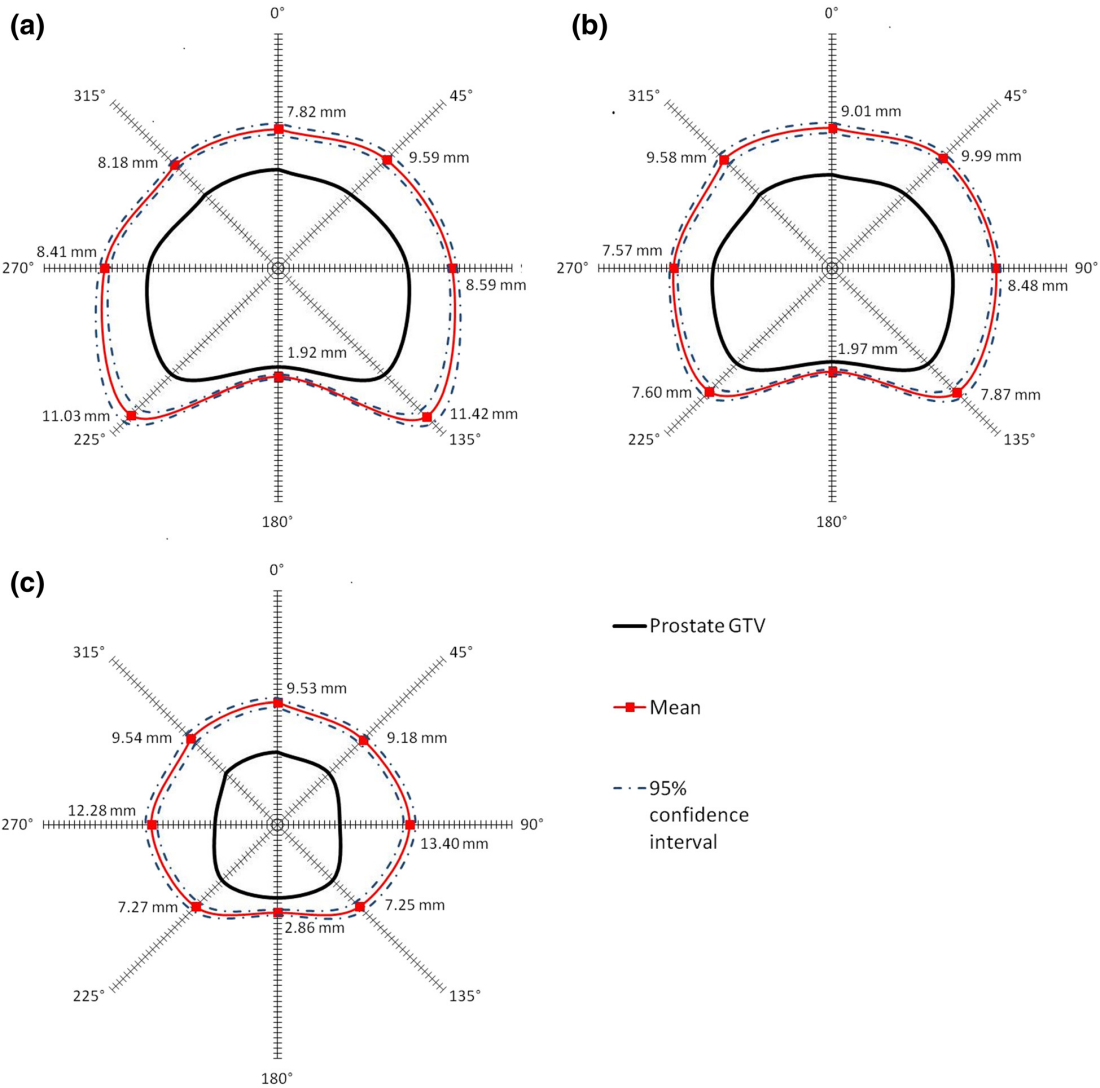
**Coverage of potential ECE.** Radar plots of the mean distance (solid red line) in millimeters of the 33 Gy isodose line from an idealized prostate GTV (solid black line). The 95% confidence interval of the mean is shown in dashed blue lines. The distances are shown on (**a**) the axial plane 1 cm caudal to base, (**b**) the axial plane in mid-prostate, and (**c**) the axial plane 0.5 cm cranial to apex.

The mean 33 Gy isodose line extends beyond 5 mm from the prostate capsule in the anterior, lateral and posterolateral directions in all 3 of the axial planes analyzed. The mean and range of these distances are listed in Table 2, along with the percent of patients who receive 33 Gy at 3, 4, 5, 6, and 7 mm on the treatment plan. The mean distances posteriorly from the prostatic capsule to the rectum are also listed in Table 2. The mean distances of the 33 Gy isodose line with the 95% confidence interval of the mean are graphed in a radar plot in Figure 2. For the majority of patients the coverage of the 33 Gy isodose line is more than 5 mm in the directions where ECE is a greater concern including the posteriolateral directions, while the extension of the high dose region into the rectum is short in comparison.

**Table 2:** Coverage of potential ECE

**Axial plane 1 cm caudal to the base**	***Mean (mm)***	***Range (mm)***	***Distance ≥ 3 mm (%)***	***Distance ≥ 4 mm (%)***	***Distance ≥ 5 mm (%)***	***Distance ≥ 6 mm (%)***	***Distance ≥ 7 mm (%)***
Anterior	7.82	1.10 – 14.42	95%	88%	85%	73%	61%
Left	8.59	4.47 – 18.46	100%	100%	95%	85%	73%
Right	8.41	4.62 – 18.15	100%	100%	95%	71%	59%
Left posterolateral	11.42	3.11 – 27.02	100%	98%	88%	83%	76%
Right posterolateral	11.03	3.12 – 27.02	100%	98%	88%	80%	73%
Posterior	1.92	−0.66 – 6.070	20%	7%	5%	2%	0%
Distance from rectum to prostate capsule	2.41	0 – 17.01					
**Axial plane at mid-prostate**	***Mean (mm)***	***Range (mm)***	***Distance ≥ 3 mm (%)***	***Distance ≥ 4 mm (%)***	***Distance ≥ 5 mm (%)***	***Distance ≥ 6 mm (%)***	***Distance ≥ 7 mm (%)***
Anterior	9.01	4.42 – 17.32	100%	100%	98%	76%	68%
Left	8.48	4.47 – 16.12	100%	100%	98%	88%	66%
Right	7.57	3.53 – 15.20	100%	98%	90%	76%	49%
Left posterolateral	7.87	2.04 – 26.86	98%	95%	83%	61%	51%
Right posterolateral	7.60	0 – 20.10	95%	85%	78%	68%	51%
Posterior	1.97	−0.66 – 4.86	29%	12%	0%	0%	0%
Distance from rectum to prostate capsule	0.87	0 – 3.58					
**Axial plane 0.5 cm cranial to apex**	***Mean (mm)***	***Range (mm)***	***Distance ≥ 3 mm (%)***	***Distance ≥ 4 mm (%)***	***Distance ≥ 5 mm (%)***	***Distance ≥ 6 mm (%)***	***Distance ≥ 7 mm (%)***
Anterior	9.53	3.56 – 15.38	100%	95%	95%	95%	83%
Left	13.40	7.64 – 22.09	100%	100%	100%	100%	100%
Right	12.28	5.55 – 21.36	100%	100%	100%	98%	95%
Left posterolateral	7.25	0 – 17.34	93%	85%	73%	61%	44%
Right posterolateral	7.27	0 – 15.81	98%	93%	85%	68%	51%
Posterior	2.86	0 – 6.83	41%	24%	12%	2%	0%
Distance from rectum to prostate capsule	0.79	0 – 3.50					

Mean distance (mm) with range between the prostatic capsule and the 33 Gy isodose line, with the percent of patients where the distance is greater than or equal to 3, 4, 5, 6, and 7 mm. The mean distance between the prostatic capsule and the rectum is also noted.

### Biochemical response

PSA values declined from a baseline mean of 7.67 ng/mL (SD 3.26 ng/mL) to a mean of 1.35 ng/mL (SD 1.08 ng/mL) at 12 months, and 0.64 ng/mL (SD 0.40 ng/mL) at 21 months. There have been no deaths from any cause to date. The 2-year Kaplan-Meier biochemical relapse-free survival rate is 97.6%. One patient experienced a biochemical failure 15 months after treatment. This patient had a prior history of obstructive urinary symptoms treated with transurethral microwave therapy prior to initiating radiotherapy. He had a baseline PSA of 6.9 ng/mL which decreased to a nadir of 2.0 ng/mL at 9 months, and then rose to 3.9 and 4.2 ng/mL in the next 2 follow-up visits while he was experiencing worsening urinary obstructive symptoms, before decreasing again to 2.1 ng/mL on subsequent follow-up. The patient was never started on ADT. Given the possibility that his rise in PSA was a benign bounce’ due to his urinary symptoms, [37] the patient is not considered to have clinically failed treatment and is currently under observation.

### Toxicity and quality of life

As previously reported, low grade urinary and rectal toxicities were common following prostate SBRT [28]. The most common urinary toxicities were frequency and urinary obstructive symptoms. The course of symptoms peaked 1–2 weeks following completion of SBRT, followed by an improvement within 2–3 months. Forty-four percent of patients experienced a late Grade 2 urinary toxicity. These correlated with alpha receptor antagonist utilization for low grade symptoms. The most common bowel toxicity was frequency of bowel movements. Seven percent of patients experienced Grade 2 late bowel toxicity secondary to rectal bleeding due to exacerbation of hemorrhoids that required ligation, banding, or laser coagulation. There were no Grade 3 or higher acute or late toxicities.

**Figure 3:**
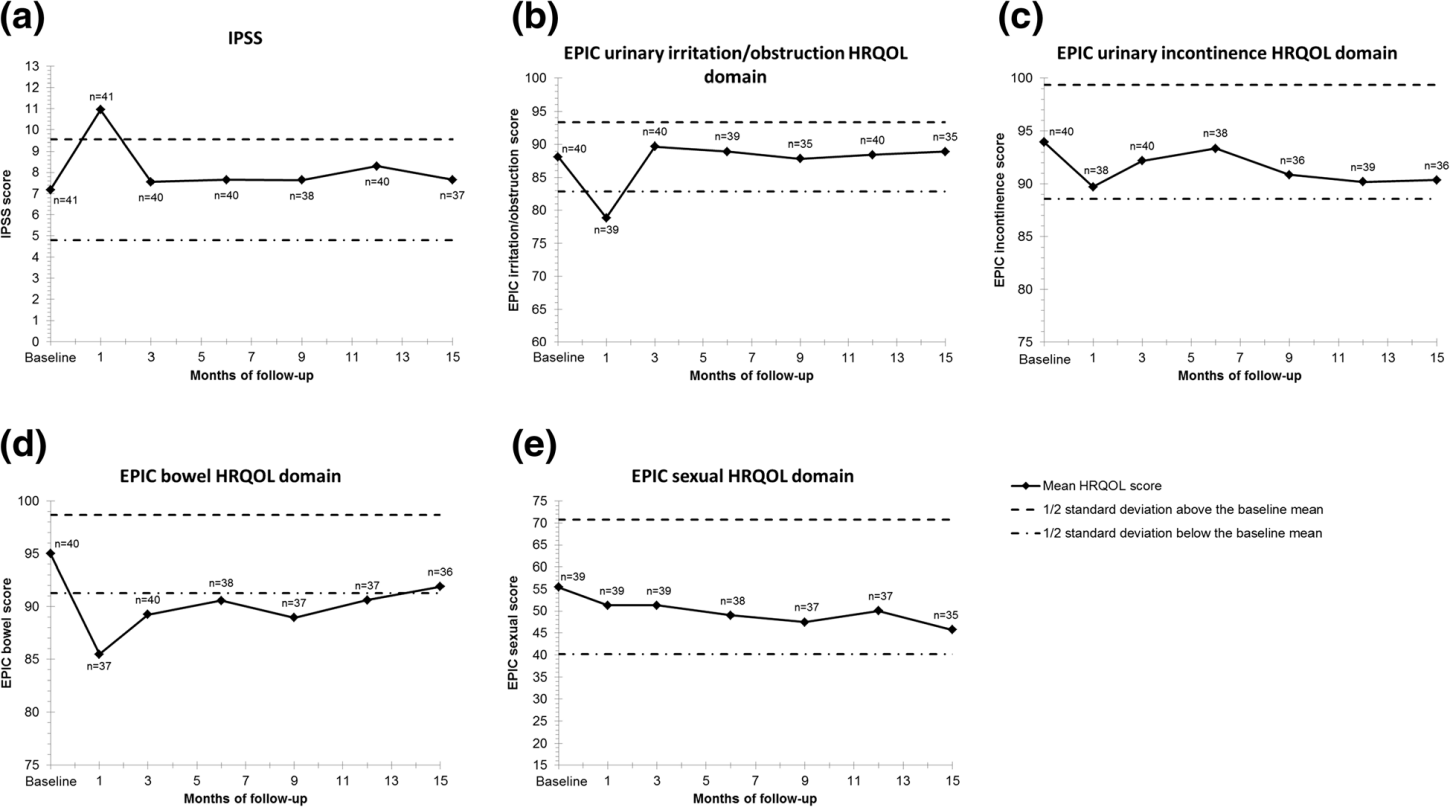
**(a-e) Mean quality of life measures at baseline and follow-up.** Analysis of the QOL data included all time points that had at least an 80% patient response rate, which was up to 15 months for all QOL measures. Shown are plots for IPSS (**a**), EPIC urinary irritation/obstruction domain (**b**), EPIC urinary incontinence domain (**c**), EPIC bowel domain (**d**), and EPIC sexual domain (**e**). The thresholds for clinically significant changes in scores (½ standard deviation above and below the baseline) are marked with dashed lines. IPSS scores range from 0–35 with higher values representing worsening urinary symptoms. EPIC scores range from 0–100 with higher values representing a more favorable health-related QOL.

Figure 3 summarizes the baseline and follow-up urinary, bowel, and sexual QOL scores. Eighty percent of patients completed their QOL questionnaires for at least 15 months. The mean IPSS (Figure 3a) and the mean EPIC urinary irritation/obstructive scores (Figure 3b) showed clinically significant (score decreased by >1/2 SD) and statistically significant (*p*<0.001 and *p*=0.035, respectively) transient decrements one month post-treatment that subsequently returned to baseline. The mean EPIC urinary incontinence score (Figure 3c) was statistically worse at 1 month (*p*=0.005), but improved by 3 months. Additional decline was observed at 9 months, but this has not reached clinical or statistical significance. The EPIC bowel score (Figure 3d) showed a clinically and statistically significant (*p*<0.001) decline at 1 month post-treatment with recovery at 15 months post-treatment. EPIC sexual scores (Figure 3e) showed a slow decline over the first year following treatment; however, the decline was not clinically or statistically significant. No clinically or statistically significant decrease occurred in the SF-12 physical or mental component QOL scores (data not shown).

## Discussion

Although IMRT is the standard external beam modality for clinically localized prostate cancer, hypofractionated SBRT monotherapy has recently emerged as a potential alternative [18]. The studies that have the longest follow-up use the CyberKnife machine for SBRT delivery, but prostate SBRT can also be delivered using other systems that account for intrafraction motion, such as the electromagnetic Calypso system or a transperineal ultrasound system. Multiple single institution experiences with SBRT monotherapy in favorable risk patients use a regimen of 35–40 Gy is delivered to the prostate in 4–5 fractions. Their results suggest that this approach may provide similar clinical outcomes to IMRT, and report high biochemical control rates with acceptable toxicity [5,16-24]. Recent updates have confirmed a 5-year biochemical disease-free survival in low-risk patients is in excess of 90% [17,21].

These SBRT monotherapy studies include mostly low-risk patients, and there is limited data on the use of hypofractionated radiotherapy in intermediate-risk patients, where ECE is more common and the treatment margins may not adequately cover this extraprostatic disease.

Pathologic reviews of post-prostatectomy specimens show that the extent of microscopic ECE in intermediate-risk patients is generally small (2–3 mm) and a 4 to 5 mm margin around the prostatic capsule should cover this spread of disease in an estimated 90-99% of intermediate-risk patients [25,33,38-41]. We consider 33 Gy (94% of the prescription dose for 35 Gy plans or 91% of the 36.25 Gy plans) to be an adequate dose for treating microscopic disease. In our study, the mean distance of the 33 Gy isodose line on the treatment plans extends >5 mm beyond the prostate capsule in all directions except directly posteriorly into the rectum, with generous coverage in the posterolateral directions where ECE more commonly occurs.

The actual dose delivered to the surrounding margin of the prostate can be less than the planned dose due to intrafraction motion of the prostate. Xie *et al.* calculated that orthogonal imaging every 30–60 seconds would allow 95.6% and 92.5% of the beams to be delivered within 2 mm of the target during CyberKnife treatments of the prostate [7]. After taking this 1–2 mm of uncertainty into account, the ECE coverage with CyberKnife SBRT is comparable to the coverage achieved in low-dose rate prostate brachytherapy. The average distance from the prostatic capsule of the 33 Gy isodose line in our study in the posteriolateral direction was 11.23 mm at the base, 7.74 mm at the mid-prostate, and 7.26 mm at the apex. This is comparable to the Merrick *et al.* study, where the distances from the prostate to the 90% isodose line in their series of Pd-103 implants as 8.4 mm at the base, 5.9 mm at the mid-prostate, and 6.8 mm at the apex [34]. The coverage directly posteriorly towards the rectum is less than in other directions, but the extent of ECE is smallest in this direction, where the rectoprostatic fascia limits the extent of invasion [40].

The mean pre-treatment PSA was 7.67 ng/mL and it decreased to a mean of 1.35 ng/mL by one year post-treatment. PSA data from patients treated with conventional external beam radiation therapy suggest that patients with PSA nadirs < 2 ng/ml at one year following treatment have a high rate of long-term disease control, [42,43] and we could predict a similarly high rate of long-term control in the patients treated in our series. Given that the majority of biochemical failures for intermediate-risk patients occur several years after treatment, the median follow-up of 21.5 months in the current study is inadequate to establish the long-term efficacy of CK monotherapy in intermediate-risk patients. However, our 97.6% 2-year biochemical failure-free survival rate is consistent with the limited literature on hypofractionated radiotherapy for intermediate-risk disease. For HDR monotherapy, which the dose distribution of SBRT monotherapy is based on, the two studies that report the biochemical control rate specifically for intermediate-risk patients show excellent 5-year PSA failure-free rates of 93% in Yoshioka *et al.* and 94% for Rogers *et al*. [14,15] In the fractionated SBRT monotherapy literature, only Lee *et al.*[24], Bolzicco *et al.*[16], Katz *et al.*[20] included a large percentage of intermediate-risk patients. Lee *et al.* report their series of 29 patients treated with SBRT to a dose of 35 to 37.5 Gy in 5 fractions. Nineteen of these patients had intermediate-risk disease, the others had low or high risk disease. The 4-year biochemical relapse-free survival was 86%. There were no Grade 3–4 acute toxicities, and one patient experienced a late Grade 3 urinary toxicity. These results are encouraging, but the results of the intermediate-risk cohort are not reported independently. Katz *et al.* reported a separate biochemical failure rate for their 81 intermediate-risk patients of 0%, but the follow-up period of their study was short, with a range of 8 to 37 months for all the patients in the study. Longer follow-up is needed to validate the observations made in our study and prospective studies are necessary for comparison to the reported 70-80% 10-year biochemical failure-free survivals obtained using conventionally fractionated external beam radiotherapy and low dose-rate brachytherapy [4].

Our study suggests that clinically significant late Grade 3 toxicities are infrequent following CK monotherapy, and our low rate is comparable to the rates observed following external beam radiotherapy, [44] HDR brachytherapy, [10,11,13,15] or in other reported CK monotherapy series [8,16,17,21-23]. Our practice allows for prescribing selective alpha blockers for relatively minor urinary complaints, which may contribute to the higher rate of recorded Grade 2 late urinary toxicity seen in our series compared to the rates reported in these other series. However, this increased higher rate of recorded low grade urinary toxicity is not reflected in our QOL results.

The QOL data indicate that CK monotherapy is well tolerated, with declines in patient reported urinary, bowel and sexual function that are similar to those seen in low-risk patients treated with CK SBRT monotherapy, [28] and comparable to the trends seen with conventionally fractionated external beam radiotherapy and brachytherapy [45-47]. Urinary symptoms peaked in the 1–2 weeks after SBRT, and were generally followed by improvement to pre-treatment levels within 2–3 months. Bowel function declined after treatment and slowly recovered to near baseline at one year. As reported by others, sexual QOL declined slowly over time without recovery [47]. Longer follow up is needed to fully assess the late impact of CyberKnife monotherapy on QOL.

## Conclusions

This analysis assesses the dosimetric feasibility and early clinical outcomes of hypofractionated stereotactic body radiotherapy delivered with the CyberKnife as monotherapy for intermediate-risk prostate cancer patients. On dosimetric evaluation, the treatment plans provide adequate coverage of potential extracapsular extension of disease and limits the dose to the adjacent rectum. Our early results show that the PSA response, absence of Grade 3 toxicity, and favorable QOL support SBRT delivered as CyberKnife monotherapy as a safe and potentially effective treatment for intermediate-risk prostate cancer. Longer follow-up is needed to more accurately assess late toxicities and biochemical failure rates of SBRT monotherapy in this patient population.

## Abbreviations

(SBRT): Stereotactic body radiation therapy; (SV): Seminal vesicles; (EBRT): External-beam radiotherapy; (HDR): High dose-rate; (CK): CyberKnife; (ECE): Extracapsular extension; (NCCN): National Comprehensive Cancer Network; (ADT): Androgen deprivation therapy; (GTV): Gross tumor volume; (CTV): Clinical target volume; (PTV): Planning target volume; (IPSS): International Prostate Symptom Score; (DRE): Digital rectal exam; (QOL): Quality of life; (EPIC): Expanded Prostate Cancer Index Composite; (SF-12): Medical Outcomes Study Short Form; (CTCAE): Common Terminology Criteria for Adverse Events; (SD): Standard deviation; (MID): Minimal important difference; (IMRT): Intensity modulated radiotherapy.

## Competing interests

The Department of Radiation Medicine at Georgetown University Hospital receives an educational grant from Accuray to support a research coordinator. Dr. Sean Collins is a clinical consultant for Accuray.

## Authors’ contributions

AJ was the lead author, who participated in the design of the project, oversaw and participated in the data collection and data analysis, and who wrote the primary drafts. HW is a biostatistician who participated in the statistical analysis and revised the statistical sections of the paper. EO aided in the quality of life data collection and statistical analysis. BS aided in the clinical data collection. SU aided in the quality of life data collection. VC aided in the quality of life data collection. AP aided in the clinical data collection. HH and JK jointly maintained the patient database, aided in data collection, and participated in initial data interpretation. SL is the dosimetrist who developed the majority of the patients’ treatment plans, and contributed to the dosimetric data analysis and interpretation. SS is a senior author who collected the dosimetric data, participated in its analysis, and helped draft the manuscript. JL is a senior author who aided in drafting the manuscript. AD is a senior author who aided in drafting the manuscript and revising its content. SC was the principle investigator who initially developed the concept of the study and the design, aided in data collection, and helped in revising the manuscript. All authors read and approved the final manuscript.
